# The Moderating Role of the Five-Factor Model of Personality in the Relationship between Job Demands/Resources and Work Engagement: An Online Cross-Sectional Study

**DOI:** 10.3390/bs14100936

**Published:** 2024-10-12

**Authors:** Toshiki Fukuzaki, Noboru Iwata

**Affiliations:** 1 Department of Clinical Psychology, Graduate School of Medical Sciences, Tottori University, Yonago 683-8503, Japan; 2 Psychosocial Epidemiology, Graduate School of Nursing, Dokkyo Medical University, Mibu 321-0293, Japan; n-iwata@dokkyomed.ac.jp

**Keywords:** five-factor model, interaction, moderating role, personality, work engagement, work environment

## Abstract

When organizations or managers utilize personality assessments for their workers, it is crucial to consider not only personality profiles but also the interaction between these profiles and the psychosocial environmental factors in the workplace. The present study aimed to examine the moderating effects of the five-factor model (FFM) of personality traits on the relationship between job demands/resources and work engagement (WE). A cross-sectional online survey was conducted between November and December 2022, targeting full-time workers in Japan. Data were collected from 1500 participants (757 men and 743 women). The survey included demographic variables, job demands and resources (job control, supervisor, and coworker support), WE, and the FFM. The primary statistical analysis was hierarchical regression analysis, which tested the interactions between job demands/resources and each personality trait. Four significant interactions were found: job demands and neuroticism, control and neuroticism, control and conscientiousness, and supervisor support and extraversion. High conscientiousness was associated with higher WE when job control was abundant. Moreover, low levels of both neuroticism and extraversion were linked to higher WE. The results suggest that managers can enhance WE by aligning workplace factors with employee personality traits. These insights can be applied to organizational staffing decisions.

## 1. Introduction

Work engagement (WE), defined as “a positive, fulfilling, work-related state of mind characterized by vigor, dedication, and absorption” [1], is used globally as an indicator of workers’ well-being [2]. Enhanced employee WE leads to better mental and physical health [3,4], higher job satisfaction, and improved job performance [5,6]. Furthermore, increased WE can lead to the formation of organizational attachment and consequently prevent turnover [7], as well as to job creativity and higher profitability for the business [8,9]. WE also enhances family life [10], contributes to better child development [11], and improves life satisfaction [12]. Consequently, various interventions have been developed to enhance WE [13,14].

According to the motivational process of the job demands–resources (JDR) model, WE increases when there are abundant job resources, such as discretionary authority, social support, and feedback [15,16,17]. This was confirmed by a recent meta-analytic review using longitudinal data [18], emphasizing the importance of creating a supportive work environment to improve WE. However, WE improves through the interplay between job resources and personal resources [18,19]. Personal resources are positive self-evaluations that indicate an individual’s ability to control and influence their environment [20], including self-efficacy, optimism, and resilience [19,21]. Focusing on both the psychosocial workplace factors and personal resources of employees could thus more effectively enhance WE [21,22].

One personal resource is personality [23,24], and research on workers’ personalities, examining its links to job satisfaction and performance, predates the concept of WE [25,26]. Recent studies have increasingly explored the relationship between WE and personality [27,28,29,30,31,32], suggesting the use of personality insights to enhance WE [33,34,35]. This study examines the relationship between the globally recognized five-factor model (FFM) of personality and WE [36].

Personality generally reflects consistent and stable patterns of thoughts and behaviors across various situations, with the FFM emerging from lexical research [37]. The FFM includes neuroticism, extraversion, conscientiousness, agreeableness, and openness, which have universal applicability across cultures [38,39,40].

Neuroticism reflects individual differences in the ease of experiencing negative emotions such as anxiety, sadness, and anger, and responses to threats [41]. High neuroticism relates to increased stress responses and poorer mental health [41,42]. Extraversion indicates an individual’s proactive engagement with the external environment, especially in interpersonal activities. Highly extraverted people are sociable, active, and seek stimulation, related to the ease of arousing positive emotions [43,44]. Conscientiousness signifies diligence, planning, and discipline, correlating with job-related indicators like income and performance [25,42]. These three factors show a consistent and strong relationship with WE compared to the other two [30,31].

Agreeableness governs systematic patterns in emotions (empathy), cognition (positive view of others), and behavior (help and cooperation) in interpersonal relations [43,45]. Highly agreeable people maintain good and warm interpersonal relationships [44]. Openness indicates broad interests, tolerance for ambiguity, and rich imagination, with highly open individuals often engaged in various intellectual activities [43,44].

Meta-analyses examining the direct relationship between the FFM and WE reveal that neuroticism shows a negative correlation while the other factors show positive correlations [30,31]. Additionally, the FFM explains around 30–40% of the variance in WE [30,31]. Therefore, addressing workers’ personalities when planning and implementing strategies to enhance WE can be effective, for instance, recruiting workers with low neuroticism and high other factors or transferring such profiles to improve WE in a particular workplace.

However, these strategies overlook the impact of psychosocial workplace factors, as they do not consider the interaction between personality and the work environment [46]. Person–environment fit theory [47] and strengths-based theory [48] argue that employees adapt better when their personality aligns well with the work environment. As such, when using personality assessments to enhance WE, it is essential to consider the combination of personality and psychosocial workplace factors.

Previous studies highlighted the usefulness of personality assessments owing to the strong direct correlation between personality traits and WE [33,34,35]. In contrast, this study focused on the indirect influence of personality traits on the relationship between work environment factors and WE. Therefore, in this study, the role of personality traits will be examined within the framework of the National Institute of Occupational Safety and Health (NIOSH) job stress model [49], considering the JDR model [15,16,17].

The NIOSH job stress model explains the relationship between stressors and acute stress reactions that arise when workers are exposed to work-related stressors [49]. Personality is considered a moderating variable that influences the relationship between stressors and acute stress reactions. This suggests that the strength of the connection between work-related stressors and acute stress reactions varies depending on the worker’s personality [50,51]. In other words, an individual’s personality may act as a moderator in the relationship between job demands, resources, and WE in the JDR model. To clarify the role of an individual’s personality as a moderator in this relationship, it is necessary to examine the interaction between job demands, resources, and each of the five personality traits in the FFM.

Generally, job demands and resources enhance WE [16,17]. Considering the interactions between job demands and resources and personality, two adjustment effects are possible: a boosting effect, where certain personality factors strengthen the relationship, and a buffering effect, where they weaken it. However, there is limited research on how job demands and resources interact with the five-factor personality traits concerning WE [52,53].

Smith and DeNunzio [52] examined full-time workers’ personality factors and interactions with coworker feedback, support, power, and autonomy, finding interactions of coworker feedback with conscientiousness or emotional stability (low neuroticism). Thus, feedback from coworkers enhances WE more in employees with high conscientiousness or high emotional stability. Hagen et al. [53] focused on job demands (job pressure and overtime) among judges, reporting interactions with conscientiousness and extraversion, where high conscientiousness and low extraversion increased WE during overtime. Fukuzaki and Iwata [54] examined the interactions between negative and positive affectivity (neuroticism and extraversion) and job demands and resources, finding no significant interactions for WE. For agreeableness and openness, we did not find any studies that reported interactions moderating the association between work environment variables related to job demands and resources and WE.

These studies vary in the workplace variables examined. Additionally, Fukuzaki and Iwata [54] combined sub-variables for job resources into a single variable, making comparisons with Smith and DeNunzio difficult [52]. In the JDR model, encompassing various variables [16,23], results vary depending on the workplace environmental factors considered. Therefore, the present study uses the job demands–control–support model [55], which is widely applicable in occupational stress research and included in the JDR model. Specifically, four variables—job demands, job control, supervisor support, and coworker support—will be used.

**Figure 1:**
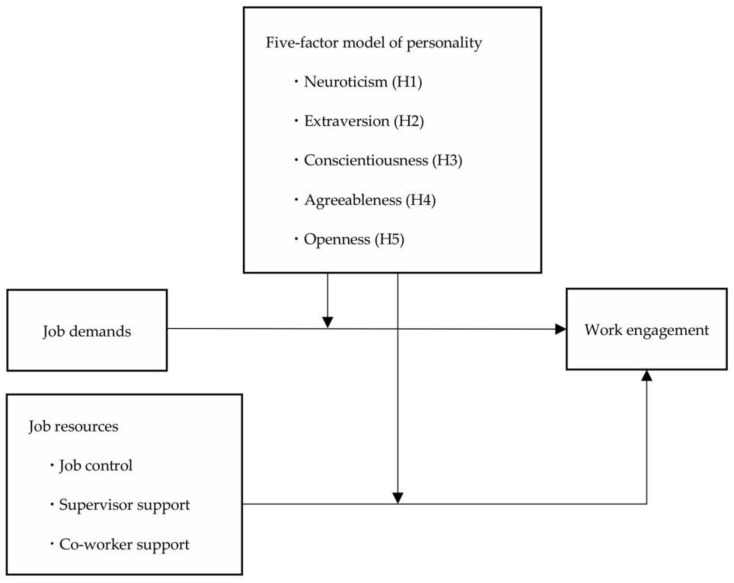
Theoretical framework for the present study.

This study aims to examine the moderating effect of the five-factor personality traits on the relationship between job demands/resources and WE (Figure 1).

The following hypotheses are proposed for each personality trait:

**Hypothesis** **1.** *Neuroticism moderates the relationship between workplace environmental factors and WE. While job demands increase WE [16,17], individuals with high neuroticism are more likely to perceive stressors as threats and experience negative emotions [41]. Therefore, individuals with high neuroticism are more likely to perceive work-related stress as a threat and are more prone to experiencing stress reactions such as tension and anxiety. Additionally, high neuroticism is associated with lower job satisfaction and performance [56]; individuals with this trait find it more difficult to experience a sense of accomplishment from completing their work [57]. Therefore, high neuroticism is believed to weaken the positive relationship between job demands and WE. In other words, individuals with emotional stability (low neuroticism) show a stronger positive relationship between job demands and WE compared to unstable individuals.*

**Hypothesis** **2.** *Extraversion moderates the relationship between workplace environmental factors and WE. Job resources have been shown to increase WE [16,17]. Additionally, individuals with high extraversion are more likely to experience positive emotions [43,44], and WE is more closely linked to positive emotions than job satisfaction [31]. In other words, a workplace rich in resources is believed to enhance WE by making it easier to work and achieve a sense of accomplishment. Furthermore, workers with high extraversion are thought to generate more positive emotions through their work, further strengthening the positive relationship between job resources and WE.*

**Hypothesis** **3.** *Conscientiousness moderates the relationship between workplace environmental factors and WE. People with high levels of conscientiousness are generally more likely to invest energy in their work [53,58] because they are diligent, disciplined, goal-oriented, and capable of planning and executing tasks systematically [42]. Because individuals with high conscientiousness are more likely to achieve results by improving their job performance [25], they can strengthen the positive relationship between job demands and WE (3-a). Similarly, they can efficiently use job resources, strengthening the positive relationship between job resources and WE (3-b) [52].*

**Hypothesis** **4.** *Agreeableness does not function as a moderator in the relationship between workplace environmental factors and WE. Agreeableness has been shown to have only a weak relationship with WE compared with other personality factors [30,31], and is generally considered a poor predictor of occupational success [45]. Teams with highly agreeable members tend to perform better because agreeableness is associated with higher empathy and the ability to build strong interpersonal relationships [43,44,59]. However, this study focuses on individuals rather than on group dynamics. Furthermore, previous research investigating the moderating effects of personality traits in the FFM on the relationship between workplace environmental factors and WE has not identified a significant buffering effect of agreeableness [52,53]. Thus, agreeableness is unlikely to serve as a moderator in this study as well.*

**Hypothesis** **5.** *Openness does not function as a moderator in the relationship between workplace environmental factors and WE. Previous research on the relationship between openness and WE has produced mixed results [30,31]. One reason for this inconsistency is that, while high openness is linked to better performance in jobs requiring creativity and intellectual exploration, this relationship tends to weaken in roles that do not demand such skills [25,60]. As the participants in this study are general workers, only a small subset likely held positions requiring high levels of creativity and exploration. Moreover, earlier studies have not found a moderating effect of openness in the relationship between workplace environment factors and WE [52,53], suggesting that openness is also unlikely to function as a moderator in this study.*

## 2. Materials and Methods

### 2.1. Participants

The survey was conducted online between November and December 2022 among registered monitors of Rakuten Insight, Inc. Rakuten Insight, Inc. is a private online research company in Japan with approximately 2.2 million registered monitors (1.3 million men and 900,000 women), and the monitors include people with a variety of attributes. Therefore, to collect data on typical Japanese workers, the screening survey excluded non-regular employees, self-employed workers, freelancers, etc. Only those who responded that they were employed by a company or organization as a regular employee proceeded to the main survey. In the main survey, the number of male and female participants was almost equal. Data were collected based on population estimates for 2021 according to Japan’s population ratio [61]. The responses were collected on a first-come, first-served basis, and data collection was terminated when the target number of 1500 respondents was attained. To minimize response errors and ensure the satisfice, we added two items from the directed questions scale (“be sure to choose the top (or bottom) option for this question”) to the survey items [62], and set it so that only those who answered correctly would have their data collected. The participants in this survey were offered a few dollars’ worth of online shopping points as an incentive. Finally, data were collected from 1500 workers (757 men and 743 women).

Table 1 presents the characteristics of the demographic variables and scale scores. The average age was 45.7 years (standard deviation 12.9) and the average length of service was 13.8 years (standard deviation 11.5). Approximately 60% of the respondents had a bachelor’s degree or higher, and approximately 60% were married. Non-manual workers accounted for approximately 70% of the occupations.

### 2.2. Measurement

#### 2.2.1. Demographic Variables

Gender, age, highest educational background, marital status, number of children, occupation, and years of employment were examined.

#### 2.2.2. Job Demands

The job demands items of the Brief Job Stress Questionnaire (BJSQ) were used [63]. This questionnaire consists of six items that ask about quantitative and qualitative job overload. These items have been shown to have acceptable internal consistency reliability (α for quantitative load = 0.77, qualitative load = 0.68) and construct validity [63], and the BJSQ is recommended as a survey form to be used for stress checks in Japan’s stress check program [64]. A typical question for quantitative overload is “I have an extremely large amount of work to do”; a typical question for qualitative overload is “I have to pay very careful attention”. Participants were asked to respond to these questions using a 4-point scale ranging from “1 = not at all” to “4 = very much so”. The total score of six items was used as the scale score (range: 6–24); the higher the scores were, the higher was the workload.

#### 2.2.3. Job Resources

Job control, supervisor support, and coworker support were measured. These variables are included in the job demand–control–support model [55]. As with the job demands, items from the BJSQ were used for these measures as well [63]. Control, supervisor support, and coworker support all consist of three items. These items showed acceptable internal consistency reliability (α for control = 0.65, supervisor support = 0.79, coworker support = 0.76) and construct validity, as did the job demands [63]. The typical question for the control is “I can work at my own pace.” The question for supervisor support and coworker support is “How freely can you talk with the following people?”; participants assume “supervisor” or “colleague” as the answer. Participants were asked to respond on a four-point scale ranging from “1 = not at all” to “4 = very much so” for the control; they were also asked to respond on a four-point scale ranging from “1 = not at all” to “4 = extremely” for support from superiors and colleagues. The total score for each was used as the scale score (range for each variable: 3–12), with higher scores indicating more discretion in work or more support from supervisors and colleagues.

#### 2.2.4. Work Engagement

The short Japanese version of the Utrecht WE scale was used [65]. WE consists of three factors—vigor, dedication, and absorption—with nine items and three items for each. The typical question for vigor is “At my work, I feel bursting with energy”; the typical question for dedication is “I find the work that I do full of meaning and purpose”; and the typical question for absorption is “Time flies when I’m working”. Respondents were asked to respond to these questions using a seven-point scale ranging from “0 = never” to “6 = always/every day”. Although three subfactors were assumed for WE, a one-factor structure is appropriate for the Japanese version [65], and it has been shown to have good internal consistency (α = 0.92) and stability reliability, factor invariance, and construct validity. Therefore, in this study, it was also treated as a one-factor structure, and the total score for all the items was used as the scale score (range: 0–54). The higher the score, the higher the WE.

#### 2.2.5. Personality Traits

The shortened version of the Big Five Personality Scale was used to measure personalities in the FFM [66]. This scale is a shortened version of the Big Five scale created by Wada based on the adjective check list [67,68] and was created using the item response theory. This scale measures 29 items in total: five items for neuroticism, five items for extraversion, seven items for conscientiousness, six items for agreeableness, and six items for openness. This scale has been shown to have good internal consistency reliability, factor structure validity, and concurrent validity comparable to the original version (α for neuroticism = 0.79, extraversion = 0.84, conscientiousness = 0.75, agreeableness = 0.77, openness = 0.77). The typical question for neuroticism is “nervous”; extraversion is “sociable”; conscientiousness is “well-planned”; agreeableness is “mild”; and openness is “versatile.” We asked the respondents to indicate how often these questions apply to them using a five-point scale ranging from “1 = not applicable at all” to “5 = entirely applicable.” The total score for each subscale was used as the scale score (range for neuroticism = 5–25, extraversion = 5–25, conscientiousness = 7–35, agreeableness = 6–30, openness = 6–30); the higher the score, the higher the respective personality trait.

### 2.3. Statistical Analysis

First, we calculated Pearson’s product–moment correlation coefficients among the following variables: age, years of employment, job demands and resources, WE, and personality. Subsequently, we conducted a hierarchical multiple regression analysis with WE as the dependent variable. The independent variables were entered in the following order. We first input the age, gender, and years of service as demographic variables (Step 1). Then, we input workplace-related factors: job demands, control, and supervisor and coworker support (Step 2). Consequently, we input the factors of five personalities (Step 3). To examine the interaction between job demands, job resources, and personality [69], we added an interaction term between job demands and personality factors (Step 4). Finally, we added interaction terms for control, supervisor and coworker support, and personality factors (Step 5). Considering multicollinearity, the interaction terms were calculated after centering using the mean. The statistical analysis was performed using R version 4.1.0.

## 3. Results

Table 2 depicts the Pearson’s product–moment correlation coefficients between each variable. WE and personality traits were all weakly correlated. Weak correlations were also identified in job control and all personality traits. All Cronbach’s alpha coefficients were above 0.70, indicating good values.

Table 3 presents the results of the hierarchical multiple regression analysis and all the entered independent variables from Step 1 to Step 5. The coefficient of determination increased significantly in all steps. The highest increase was in Step 2, which involved job demands and resources (ΔR^2^ = 0.17, ΔF(4, 1492) = 82.53, *p* < 0.001), followed by Step 3, which involved personality factors (ΔR^2^ = 0.08, F(5, 1487) = 35.66, *p* < 0.001).

The most correlated variable to WE in Step 5, where all independent variables were entered, was job demands (β = 0.17, t = 7.54, SE = 0.07, *p* < 0.001), followed by supervisor support (β = 0.15, t = 4.82, SE = 0.16, *p* < 0.001), and the third most correlated variable was conscientiousness of personality factors (β = 0.14, *t* = 5.59, SE = 0.07, *p* < 0.001). All variables including job demands, resources, and personality factors were significantly associated with WE.

**Figure 2:**
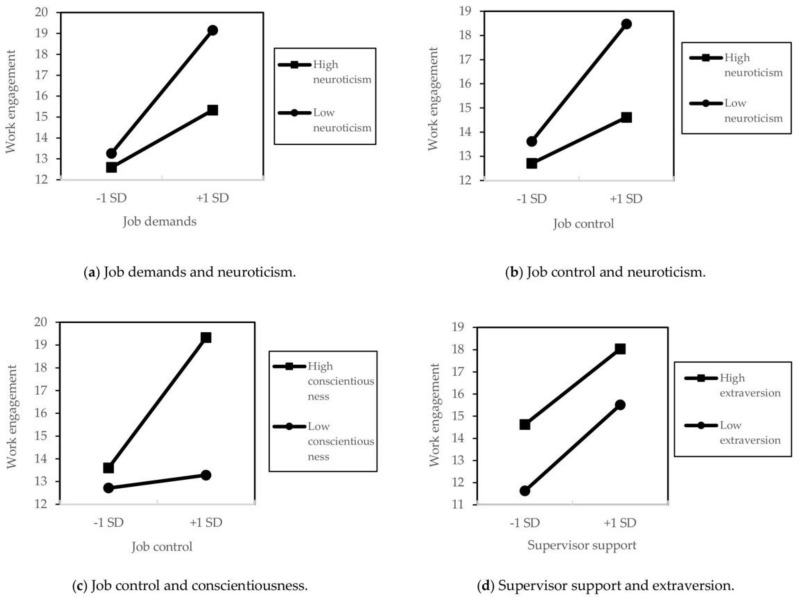
Simple slope analysis to examine the interaction effects of relationships between (**a**) job demands and neuroticism, (**b**) job control and neuroticism, (**c**) job control and conscientiousness, and (**d**) supervisor support and extraversion.

Concerning the interaction effects, four associations were significant: job demands and neuroticism (β = −0.05, *t* = −2.25, SE = 0.02, *p* < 0.05), control and neuroticism (β = −0.05, *t* = −2.08, SE = 0.04, *p* < 0.05), control and conscientiousness (β = 0.10, *t* = 3.64, SE = 0.03, *p* < 0.001), and supervisor support and extraversion (β = −0.08, *t* = −2.08, SE = 0.05, *p* < 0.05). A simple slope analysis was performed in these associations. The results depicted that, regardless of the level of neuroticism, WE increases as job demands or control increase, but increases more when neuroticism is low (Figure 2a,b). Subsequently, the results indicated that WE does not change even if the control is high when conscientiousness is low, but WE increases when conscientiousness is high (Figure 2c). Finally, the results indicated that WE increases with greater supervisor support regardless of the level of extraversion, but WE increases more when extraversion is low (Figure 2d).

## 4. Discussion

The purpose of this study was to examine the moderating effects of the FFM of personality traits on the relationship between job demands/resources and WE. The results showed four significant interaction effects: neuroticism with job demands (Hypothesis 1), neuroticism with job control, extraversion with supervisor support (Hypothesis 2), and conscientiousness with job control (Hypothesis 3-b). No significant interactions were found for agreeableness or openness (Hypotheses 4 and 5). Thus, Hypotheses 1, 3-b, 4, and 5 were supported, while Hypotheses 2 and 3-a were not. Notably, the interaction between extraversion and job resources showed that individuals with lower extraversion had higher WE, contrary to the hypothesis. Additionally, the significant interaction between neuroticism and job control was unexpected.

First, all personality traits were significantly correlated with WE in both Pearson’s correlation and hierarchical regression analyses, consistent with previous meta-analyses [30,31]. Furthermore, in Step 5 of the hierarchical regression analysis, where all variables were included, the standardized regression coefficients for the FFM traits remained significant, underscoring the importance of considering workers’ personalities to enhance WE [28,29,33].

In the interaction between conscientiousness and job control, low conscientiousness showed no change in WE, regardless of the level of control. However, when conscientiousness was high, there was a boosting effect, with WE increasing as control increased (Hypothesis 3-b). However, conscientiousness did not moderate the relationship between job demands and WE (Hypothesis 3-a). As mentioned earlier, conscientiousness reflects traits such as diligence, planning, and discipline [42,44]. Studies have shown that individuals with high conscientiousness invest more energy in their work [53,58], leading to improved performance [25]. When workers with high conscientiousness are given substantial discretion, they are likely to use it to work diligently and systematically, leading to greater WE and earning respect from their colleagues [70]. Conversely, workers who are not highly conscientious will likely remain unenthusiastic about their work, even with a high level of discretion, owing to their lack of seriousness, discipline, and sense of responsibility [42,44].

Neuroticism was found to interact with two other factors: job demands and job control. The results suggest that when workload and discretionary authority are high, WE increases regardless of the level of neuroticism; however, the increase is more pronounced for those with low neuroticism. Conversely, the interaction effect of extraversion is related only to supervisor support. This finding indicates that when supervisor support is high, WE increases regardless of the level of extraversion; however, the increase is greater for workers with low extraversion than for those with high extraversion. High levels of WE are typically associated with high extraversion and low neuroticism [27]. Additionally, when comparing the direct relationship between WE and either neuroticism or extraversion, some studies reported that extraversion has a stronger association [30], while others reported similar levels [31]. Regarding the interaction between neuroticism, extraversion, and workplace environmental factors, this study found that low neuroticism was associated with a higher level of WE than high extraversion, suggesting that emotional stability (low neuroticism) is more crucial than high extraversion for enhancing WE.

The interaction between neuroticism and job demands supported hypothesis 1, showing that WE increased more for workers with low neuroticism. Highly neurotic individuals perceive even mundane situations as threats and are easily overwhelmed by minor frustrations [41]. Additionally, they tend to view their work environment negatively and struggle to experience a sense of accomplishment [57,71,72]. However, emotional stability mitigates the negative impacts of high job demands [73,74]. Therefore, emotionally stable workers gain vitality from their work, whereas highly neurotic workers are likely to feel threatened even by low-stress tasks; because they struggle to feel a sense of accomplishment, the energy they derive from their work is thought to be diminished.

The significant interaction between neuroticism and job control was unexpected but can be interpreted similarly to its interaction with job demands. For example, a manager with substantial discretionary power may need to make important decisions that shape the direction of their department or organization. Additionally, having a high level of discretion often requires handling a wide range of tasks, from administrative duties to hands-on work. At such times, workers with a high tendency toward neuroticism may view their workload negatively and may assess themselves, the organization that entrusted them with discretion, and their managers unfavorably [71,72]. Neurotic tendencies are thought to undermine the positive relationship between job control and WE. Conversely, because workers with low neurotic tendencies and greater emotional stability show increased engagement, these results further underscore the importance of emotional stability.

In the interaction between extraversion and job resources (Hypothesis 2), a reverse relationship was observed with supervisor support. Numerous previous studies have highlighted the importance of extraversion as a key personality factor, noting its general positive correlation with WE [27,28,30,31]. However, our results revealed a buffering effect in the interaction with supervisor support, indicating that higher extraversion scores weakened the relationship between supervisor support and WE. It has been suggested that extraverted employees may become more sociable when receiving support, leading to distractions and decreased focus on work [52]. Highly extraverted individuals can proactively seek support when needed; thus, passive and excessive support from supervisors can potentially hinder their work. In other words, support from superiors may be more effective in enhancing the engagement of workers with low extraversion.

The results above not only provide new insights into recruiting individuals with personality profiles likely to enhance WE, but also highlight the importance of aligning personalities with the workplace environment to boost WE. Research has shown that when employees with high engagement are in the same department, the crossover effect can increase the engagement of the other employees [32,75]. Therefore, if an organization aims to increase WE in a specific department, personnel allocation that considers the match between personality profiles and workplace environment factors may be effective. For instance, assigning individuals with low neuroticism and high conscientiousness and extraversion to a department, while granting them considerable discretion over their work, can create an environment conducive to high WE and generate a positive ripple effect on those around them.

However, it is crucial to use personality assessments cautiously, as different variables interact with job demands/resources, potentially increasing or decreasing engagement. Because personalities are influenced by changes in the work environment [76], it may be beneficial to regularly assess them, just as WE and work environment factors are evaluated. Rubenstein et al. [77] suggest that incorporating personality assessments into recruitment can be cost-effective for improving employees’ perceptions and attitudes toward their work environment in the long term. Thus, personality assessments could have potential value as a new intervention to enhance WE.

This study has several limitations. First, all the participants were registered monitors selected by the same online survey company. Despite collecting data proportional to Japan’s population ratio, the selection bias from using the same survey company might have influenced the results. Future research should include non-internet users. Second, this study was cross-sectional, preventing the examination of causal relationships between job demands/resources, personality, and WE. Longitudinal studies are needed to explore these causal relationships, as workplace factors can also influence personality [76,78]. Third, only one variable, job demands, was treated in this study. As there are various characteristics of job demands as well as job resources in previous studies [79], it is necessary to examine the interaction between them and personality. Finally, this study did not account for the impact of job types. Different jobs might have different demands and resources, and specific personality traits may be predominant in certain job types (e.g., salespeople might have high extraversion). Considering job type impacts when examining interactions between workplace factors and personality could yield more detailed insights.

## 5. Conclusions

This study confirmed four interactions between the FFM of personality and psychosocial workplace factors, highlighting the effectiveness of considering both personality and workplace factors to enhance WE. These insights can be utilized in organizational staffing.

## Figures and Tables

**Table 1:** Demographic characteristics of respondents (N = 1500).

	N	%
**Gender**		
Men	757	50.5
Women	743	49.5
**Age (years)**	45.7 ^1^	12.9 ^2^
**Education**		
University/graduate school graduate	873	58.2
Vocational school/college graduate	354	23.6
High school graduate	263	17.5
Others	10	0.7
**Marital status**		
Unmarried	488	32.5
Married	833	55.5
Divorce/bereavement	179	11.9
**Number of children**		
0	708	47.2
1	265	17.7
2	362	24.1
3≥	165	11.0
**Occupation**		
Manager	237	15.8
Non-manual worker	1057	70.5
Manual worker	78	5.2
Other	128	8.5
**Career in the current job (years)**	13.8 ^1^	11.5 ^2^

^1^ Mean. ^2^ SD.

**Table 2:** Correlations and reliability estimates (Cronbach’s alpha) (N = 1500).

	Mean	SD	1	2	3	4	5	6	7	8	9	10	11	12
1. Age	45.7	12.9	―											
2. Career in the current job	13.8	11.5	0.55 ***	―										
3. Job demands	16.4	4.0	−0.08 **	−0.03	(0.87)									
4. Job control	8.0	2.1	0.13 ***	0.11 ***	−0.01	(0.79)								
5. Supervisor support	7.4	2.4	−0.02	−0.01	0.02	0.30 ***	(0.90)							
6. Co-worker support	7.8	2.3	−0.01	0.01	0.06 *	0.24 ***	0.69 ***	(0.88)						
7. Work engagement	22.1	12.4	0.21 ***	0.09 ***	0.17 ***	0.29 ***	0.31 ***	0.30 ***	(0.97)					
8. Neuroticism	16.1	3.9	−0.21 ***	−0.08 **	0.19 ***	−0.17 ***	−0.15 ***	−0.15 ***	−0.24 ***	(0.84)				
9. Extraversion	15.4	3.8	0.09 ***	0.02	0.06 *	0.20 ***	0.22 ***	0.28 ***	0.30 ***	−0.26 ***	(0.84)			
10. Conscientiousness	22.4	4.5	0.25 ***	0.09 ***	−0.02	0.12 ***	0.05	0.03	0.28 ***	−0.29 ***	0.07 *	(0.79)		
11. Agreeableness	19.3	3.6	0.12 ***	0.02	0.02	0.14 ***	0.15 ***	0.18 ***	0.27 ***	−0.27 ***	0.23 ***	0.35 ***	(0.75)	
12. Openness	18.2	3.9	0.09 ***	0.01	0.08 **	0.23 ***	0.16 ***	0.14 ***	0.25 ***	−0.12 ***	0.50 ***	0.15 ***	0.26 ***	(0.81)

* *p* < 0.05, ** *p* < 0.01, *** *p* < 0.001.

**Table 3:** Hierarchical multiple regression analysis to test the interaction effects of job demand and resources, five factors of personality on work engagement (N = 1500).

	B ^1^	95%CI ^2^	β ^3^	ΔR^2 4^	Adjusted R^2 4^
**Step 1**				0.05	0.04 ***
Age	0.13	0.08–0.18	0.13 ***		
Gender (0 = Men, 1 = Women)	1.14	0.05–2.23	0.05 *		
Career in the current job	−0.01	−0.07–0.04	−0.01		
**Step 2**				0.17 ***	0.22 ***
Job demands	0.52	0.38–0.65	0.17 ***		
Job control	0.71	0.44–0.99	0.12 ***		
Supervisor support	0.76	0.45–1.08	0.15 ***		
Co-worker support	0.39	0.06–0.72	0.07 *		
**Step 3**				0.08 ***	0.30 ***
Neuroticism	−0.28	−0.43–(−0.13)	−0.09 ***		
Extraversion	0.35	0.18–0.53	0.11 ***		
Conscientiousness	0.38	0.25–0.52	0.14 ***		
Agreeableness	0.26	0.10–0.43	0.08 **		
Openness	0.19	0.02–0.35	0.06 *		
**Step 4**				0.01 *	0.30 ***
Job demands × Neuroticism	−0.04	−0.07–(−0.01)	−0.05 *		
Job demands × Extraversion	−0.00	−0.05–0.04	−0.01		
Job demands × Conscientiousness	0.03	−0.00–0.06	0.04		
Job demands × Agreeableness	−0.02	−0.06–0.02	−0.02		
Job demands × Openness	0.01	−0.03–0.04	0.01		
**Step 5**				0.02 ***	0.31 ***
Job control × Neuroticism	−0.07	−0.14–(−0.00)	−0.05 *		
Job control × Extraversion	−0.01	−0.09–0.07	−0.01		
Job control × Conscientiousness	0.11	0.05–0.18	0.10 ***		
Job control × Agreeableness	−0.01	−0.09–0.07	−0.01		
Job control × Openness	−0.02	−0.09–0.05	−0.02		
Supervisor support × Neuroticism	0.04	−0.04–0.12	0.04		
Supervisor support × Extraversion	−0.10	−0.20–(−0.01)	−0.08 *		
Supervisor support × Conscientiousness	0.05	−0.02–0.12	0.05		
Supervisor support × Agreeableness	0.04	−0.05–0.13	0.03		
Supervisor support × Openness	−0.01	−0.09–0.08	−0.01		
Co-worker support × Neuroticism	−0.01	−0.10–0.07	−0.01		
Co-worker support × Extraversion	0.08	−0.02–0.18	0.06		
Co-worker support × Conscientiousness	0.00	−0.07–0.08	0.00		
Co-worker support × Agreeableness	−0.08	−0.17–0.02	−0.06		
Co-worker support × Openness	0.06	−0.03–0.15	0.05		

^1^ B = unstandardized regression coefficients. ^2^ CI = confidence interval. ^3^ β = standardized regression coefficients. ^4^ R^2^ = coefficient of determination. * *p* < 0.05; ** *p* < 0.01; *** *p* < 0.001.

## Data Availability

The data presented in this study are not publicly available but are available from the corresponding author on reasonable request.
